# Treatment and prophylaxis of melioidosis

**DOI:** 10.1016/j.ijantimicag.2014.01.005

**Published:** 2014-04

**Authors:** David Dance

**Affiliations:** a Lao-Oxford-Mahosot Hospital-Wellcome Trust Research Unit (LOMWRU), Microbiology Laboratory, Mahosot Hospital, Vientiane, Lao People's Democratic Republic; b Centre for Tropical Medicine, Nuffield Department of Medicine, University of Oxford, Oxford, UK

**Keywords:** Melioidosis, *Burkholderia pseudomallei*, Treatment, Prophylaxis, Antibiotics

## Abstract

Melioidosis, infection with *Burkholderia pseudomallei*, is being recognised with increasing frequency and is probably more common than currently appreciated. Treatment recommendations are based on a series of clinical trials conducted in Thailand over the past 25 years. Treatment is usually divided into two phases: in the first, or acute phase, parenteral drugs are given for ≥10 days with the aim of preventing death from overwhelming sepsis; in the second, or eradication phase, oral drugs are given, usually to complete a total of 20 weeks, with the aim of preventing relapse. Specific treatment for individual patients needs to be tailored according to clinical manifestations and response, and there remain many unanswered questions. Some patients with very mild infections can probably be cured by oral agents alone. Ceftazidime is the mainstay of acute-phase treatment, with carbapenems reserved for severe infections or treatment failures and amoxicillin/clavulanic acid (co-amoxiclav) as second-line therapy. Trimethoprim/sulfamethoxazole (co-trimoxazole) is preferred for the eradication phase, with the alternative of co-amoxiclav. In addition, the best available supportive care is needed, along with drainage of abscesses whenever possible. Treatment for melioidosis is unaffordable for many in endemic areas of the developing world, but the relative costs have reduced over the past decade. Unfortunately there is no likelihood of any new or cheaper options becoming available in the immediate future. Recommendations for prophylaxis following exposure to *B. pseudomallei* have been made, but the evidence suggests that they would probably only delay rather than prevent the development of infection.

## 1 Introduction

Melioidosis is the name given to any infection caused by the saprophytic environmental bacterium *Burkholderia pseudomallei*, which is widespread in the soil and surface water in southeast Asia and northern Australia. The disease is being recognised with increasing frequency in known endemic areas [1], [2] and new foci are regularly being identified [3], [4].

The organism is intrinsically resistant to many antimicrobial agents, including those often used for the empirical treatment of sepsis in the tropics [5], and may be even more resistant when growing in biofilms [6], [7], [8] and in the anaerobic acidic conditions that might be found in vivo [9]. There is considerable evidence supporting current treatment recommendations, mainly derived from a series of large randomised clinical trials conducted in northeast Thailand since 1986, although there are also many unanswered questions. This review will summarise that evidence and the current recommendations and will consider some of the outstanding issues.

## 2 Treatment

The current convention is to view the treatment of melioidosis as comprising two phases: the first is the acute phase, the aim of which is to stop patients from dying of overwhelming sepsis; the second is the eradication phase, the aim of which is to kill any residual bacteria and to minimise the risk of the infection relapsing.

### 2.1 Acute phase (Table 1)

Until 1985, the usual treatment for the acute phase was a combination of chloramphenicol, doxycycline and trimethoprim/sulfamethoxazole (co-trimoxazole) (the ‘conventional’ regimen). However, the overall mortality was 37.9–61% and for patients with septicaemic infection and multiple foci it was as high as 87% [10]. Since there were new β-lactams available that showed promising in vitro activity against *B. pseudomallei* [11], the first of several randomised prospective clinical trials of treatment for melioidosis was started in Ubon Ratchathani, northeast Thailand, in 1986 [12]. Patients were randomised to receive the conventional regimen or ceftazidime (120 mg/kg/day) according to a paired restricted sequential design. In total 161 patients were entered into the study, of whom 65 had culture-proven melioidosis and 54 of these were septicaemic. The overall mortality was 37% in those treated with ceftazidime compared with 74% in the conventionally treated group, a reduction of 50% [95% confidence interval (CI) 19–81%], suggesting that ceftazidime should be adopted as the acute-phase treatment of choice for severe melioidosis. Mortality in patients with septicaemic melioidosis was reduced from 76% to 43%, and in patients in whom melioidosis was not confirmed from 79% to 61%. Ceftazidime was given for a median of 8 days (range 7–28 days). Despite these encouraging results, it is worth noting that 4/20 patients and 1/9 patients still had positive blood cultures on Days 3 and 7 of treatment, respectively, and that 3 patients subsequently had bacteriologically confirmed relapses of melioidosis, indications of the recalcitrant nature of the disease.

A similar study, which also took place in northeast Thailand, was reported 3 years later [13] with broadly similar results. The study design differed in that ceftazidime was given at a slightly lower dose (100 mg/kg/day) combined with co-trimoxazole. The reported 7-day mortalities amongst the 61 evaluable patients with confirmed melioidosis in that study were lower than in the previous study (47% with conventional treatment compared with 18.5% in the ceftazidime plus co-trimoxazole group overall; 57.7% compared with 25% for septicaemic melioidosis; and 82.3% compared with 30.7% for disseminated septicaemic melioidosis). No significant difference in mortality was found in patients with established septic shock at the time of presentation. It was also reported that no relapses were seen amongst survivors in that study.

Whether the differences between the two studies described above reflected a genuinely better outcome with the combination than with ceftazidime monotherapy, or differences in the severity of illness between patients included in the two studies, was initially unclear. Two studies comparing ceftazidime with and without co-trimoxazole undertaken in Khon Kaen and Ubon Ratchathani were subsequently published as a single paper [14]. The overall in-hospital mortality rates amongst all 449 patients enrolled were not significantly different between those treated with ceftazidime alone (25.1%) and those treated with the combination (26.6%), nor were there differences in death rates amongst the 241 patients with culture-confirmed melioidosis, either overall or occurring ≥48 h after admission. Multiple logistic regression analysis identified that bacteraemia, respiratory failure and renal failure, but not drug regimens, were independently associated with death and treatment failure, even when cases with co-trimoxazole-‘resistant’ isolates were excluded. On prolonged follow-up, there was also no difference between the two groups in terms of mortality or culture-confirmed recurrence [15]. Thus, there is no evidence to support the routine addition of co-trimoxazole to ceftazidime during the acute phase of treatment for melioidosis, although some have argued that this is warranted in patients with undrained deep-seated infections or when monotherapy fails in places where carbapenems are unavailable or unaffordable [16].

In an effort both to reduce the cost of treatment and to evaluate an agent with a spectrum of activity that might be more appropriate for monotherapy of community-acquired sepsis than ceftazidime alone, amoxicillin/clavulanic acid (co-amoxiclav) (160 mg/kg/day) was compared with ceftazidime (120 mg/kg/day) for treatment of severe melioidosis in Ubon Ratchathani in a large, open, paired randomised controlled trial between 1989 and 1992 [17]. In total, 379 patients were enrolled, of whom 212 proved to have culture-positive melioidosis, with 106 patients in each treatment arm. There were no significant differences in mortality between the two groups (overall 47%). The study design allowed the treating physicians to switch treatment if the clinical response was considered ‘unsatisfactory’ after ≥72 h, however, and this occurred more frequently in the co-amoxiclav group (16/69) than in the ceftazidime group (4/75). This is clearly a somewhat subjective endpoint, but as a result ceftazidime was considered to remain the treatment of choice, with co-amoxiclav as a second-line option.

The nature of the mixture of amoxicillin and clavulanic acid means that there are complex pharmacokinetic considerations when using it to treat melioidosis. For example, there is in vitro evidence that relatively high concentrations of clavulanic acid must be achieved to potentiate amoxicillin [18], and modelling suggests that the dosing interval for co-amoxiclav in melioidosis should not be >6 h [19]. Since different formulations and ratios of amoxicillin to clavulanic acid are available in different countries, and different regimens have been used by different groups to treat melioidosis, an international consensus statement was published in 2008 to reduce confusion [20]. This recommends the use of amoxicillin/clavulanic acid at a dose of 20/5 mg/kg every 4 h, but only as a second-line agent for acute-phase treatment.

Another β-lactam/β-lactamase inhibitor combination that has good in vitro activity against *B. pseudomallei* [minimum inhibitory concentration required to inhibit 90% of the isolates (MIC_90_) = 4 mg/L] is cefoperazone/sulbactam [21]. This was evaluated at 25 mg/kg/day in combination with co-trimoxazole (trimethoprim 8 mg/kg/day) in comparison with ceftazidime (100 mg/kg/day) plus co-trimoxazole [22]. In total, 219 patients were enrolled, of whom 102 had culture-confirmed melioidosis. There were no significant differences in mortality between the two groups (18% compared with 14%, respectively) or in fever duration or bacteriological response. This study was, however, relatively underpowered [23]. Furthermore, in a retrospective analysis of 1353 patients with melioidosis who received cephalosporins, the overall mortality rate for those who received cefotaxime or ceftriaxone (71%) was significantly higher than those receiving ceftazidime (41.7%) or co-amoxiclav (53.9%) [24]. Ceftazidime has thus remained the cephalosporin of choice for acute treatment of melioidosis [25]. There is evidence that the total dose, and therefore costs, of ceftazidime may be reduced from 120 mg/kg/day to 96 mg/kg/day if it is given by continuous infusion rather than bolus dosing [26]. To facilitate outpatient treatment with ceftazidime and to optimise pharmacokinetics, clinicians in northern Australia have used a simple elastomeric infusion apparatus to administer ceftazidime [27]. This approach can save significant expenditure on inpatient care if the infrastructure to support outpatient parenteral antibiotic therapy is available, but as yet it has not been widely adopted internationally [28]. In northern Australia there is now a trend towards using increasingly long courses of intravenous (i.v.) antibiotics to treat melioidosis, especially in the presence of deep-seated undrained foci of infection. It has been suggested that this approach may ultimately obviate the need for an eradication phase, although it has not been evaluated in comparative trials. The median duration of the i.v. phase in Darwin is now 4 weeks, and some 27% of patients have had no eradication-phase treatment without developing relapse [29].

The carbapenems are the most active drugs against *B. pseudomallei* in vitro [30]. There are also some theoretical reasons for believing that they may be better therapeutic options than ceftazidime. For example, they exhibit longer post-antibiotic effects [31] and are more rapidly bactericidal [30]. An open, prospective, randomised study was therefore conducted to compare the efficacy of ceftazidime (120 mg/kg/day) with that of imipenem/cilastatin (50 mg/kg/day) for a minimum of 10 days [32]. Unfortunately, the study had to be terminated prematurely due to the withdrawal of pharmaceutical company support, by which time 296 patients had been enrolled, of whom 214 had culture-confirmed melioidosis. There were no differences between the regimens in mortality either overall (36.1% vs. 37.7%) or after 48 h of treatment (14.8% vs. 21.4%), but the somewhat subjective outcome of ‘treatment failure’ was significantly more common in patients treated with ceftazidime (41.3% compared with 20.3%). In addition, significantly greater endotoxin release during treatment occurred in patients treated with ceftazidime [33].

Latterly, meropenem has tended to be preferred over imipenem/cilastatin as the carbapenem of choice, largely because of lower toxicity, and in northern Australia it has been used extensively in the treatment of severe melioidosis, albeit not in comparative trials. In a retrospective review of 63 patients treated with meropenem for severe melioidosis in Darwin over a 6-year period, comparable outcomes to those with ceftazidime were observed, with a mortality of only 19% despite a bias towards the more severely unwell patients receiving meropenem [34]. Unfortunately, a multicentre, randomised comparative study comparing meropenem with ceftazidime in the treatment of severe melioidosis in northeast Thailand is currently stalled.

### 2.2 Eradication phase (Table 2)

The recalcitrant nature of melioidosis with its tendency to recurrence, leading in the USA to the nickname ‘The Vietnam time bomb’, is well known. As improved treatment for the acute phase led to larger numbers of survivors, it became increasingly important to identify regimens that would reduce relapses to a minimum. Perhaps somewhat surprisingly, it was found that ca. 25% of patients with recurrent melioidosis actually have re-infections with a different strain rather than relapses of their initial infection [35]. Multivariate analysis has shown that the most important risk factors for true relapse are the agent and duration of oral treatment, followed by positive blood cultures and multifocal infection [36].

The conventional regimen (chloramphenicol plus doxycycline plus co-trimoxazole) was always associated with a high risk of side effects, and furthermore there was some in vitro evidence of the mutual antagonism of these agents [37], [38] and a risk of resistance emerging to all of them simultaneously [5]. The first comparative study of eradication treatment was started in Ubon Ratchathani in 1989 and took over 3 years to complete [39]. In total, 101 patients were randomised to receive either chloramphenicol (40 mg/kg/day in four divided doses) plus doxycycline (4 mg/kg/day in two divided doses) plus co-trimoxazole (10/50 mg/kg/day in two divided doses) or co-amoxiclav with additional amoxicillin (60 mg/kg/day amoxicillin plus 15 mg/kg/day clavulanic acid) for 20 weeks, with chloramphenicol being discontinued after the first 8 weeks to reduce the risk of toxicity. Patients infected with isolates exhibiting in vitro resistance to any of the study drugs or in whom any were contraindicated were excluded. It was shown that 8 of 49 patients in the co-amoxiclav group and 2 of 52 patients in the conventional group had culture-proven relapses, although a number of patients were lost to follow-up. Overall compliance was poor, with only one-half of the patients receiving the full 20 weeks of treatment, of whom two in each group relapsed, reflecting the difficulties of undertaking this sort of study. Relapse rates were higher in patients receiving co-amoxiclav (10%) as opposed to conventional treatment (4.9%) for >12 weeks, although none of these differences achieved statistical significance. Poor compliance was associated with a greater risk of relapse, as was multifocal disease. Co-amoxiclav was, however, better tolerated than the conventional regimen, which was associated with adverse effects in 29% of cases. The conclusion of this study was that conventional therapy was probably more effective and considerably cheaper than co-amoxiclav, which the authors estimated to cost ca. 15 times more than the conventional regimen at that time, and so the conventional regimen would therefore usually be preferred, except in children and pregnant or lactating women in whom the conventional regimen is contraindicated.

Fluoroquinolones are only marginally active in vitro against *B. pseudomallei* (MIC_50_ and MIC_90_ for ciprofloxacin = 4 mg/L) [5]. They do achieve high intracellular concentrations, however, and since *B. pseudomallei* is able to survive intracellularly and is often referred to as an intracellular pathogen (although this is a considerable oversimplification), attempts have been made to evaluate the therapeutic efficacy of this group of drugs in melioidosis. In an open pilot study of ciprofloxacin (20 mg/kg/day) and ofloxacin (12 mg/kg/day) for eradication therapy in 57 patients with melioidosis, there were 13 treatment failures (5 failures to respond and 8 relapses) [40]. This disappointing failure rate of 29% suggested that fluoroquinolones should not be used for treatment of melioidosis unless there are no alternatives. A subsequent randomised, open study conducted at two hospitals in Thailand investigated whether the addition of azithromycin, selected on the basis of its intracellular penetration and activity against biofilms despite only moderate in vitro activity against *B. pseudomallei*, might potentiate the efficacy of ciprofloxacin in the eradication phase of treatment [41]. In total, 65 patients were randomised to receive ciprofloxacin plus azithromycin for 12 weeks (32 patients, of whom 7 relapsed) or co-trimoxazole plus doxycycline for 20 weeks (33 patients of whom only 1 relapsed). This significant difference in outcomes confirms that regimens containing fluoroquinolones should not be used for treating melioidosis.

Since the original conventional treatment was a purely empirical approach comprising multiple agents, some of which are mutually antagonistic in vitro [37] and all of which can cause potentially serious side effects, several studies have been conducted to assess which components of the regimen might be omitted without adversely affecting the outcome. The first such study compared the combination of chloramphenicol, doxycycline and co-trimoxazole with doxycycline alone for a minimum of 12 weeks in an open randomised study involving 116 patients, including 109 with culture-confirmed melioidosis, of whom 87 were considered evaluable [42]. Bacteriological relapse occurred in only one patient treated with the four-drug combination but in 11 patients who received doxycycline alone, and treatment failure was judged to have occurred in 18.2% and 46.5%, respectively, suggesting that doxycycline alone was inadequate eradication therapy for melioidosis.

In a subsequent open-label randomised study, the effect of omitting chloramphenicol, potentially the most toxic component of the conventional oral regimen, was evaluated [43]. In total, 180 patients were randomised to receive the oral regimen with (91 patients) or without (89 patients) chloramphenicol for the first 4 weeks of 12–20 weeks of total treatment. The trial was terminated early due to poor drug tolerance, particularly of the four-drug regimen (36% requiring a switch in therapy due to side effects compared with 19%), but no significant difference was found between the relapse rates with the two regimens (6.6% and 5.6%, respectively), confirming that chloramphenicol could, or rather should, be omitted. It was noted, however, that patients who received <12 weeks of oral therapy had a 5.7-fold increased risk of relapse or death, emphasising the importance of compliance with prolonged oral treatment. It is vital that this is clearly and carefully explained to patients at every opportunity.

There remains some uncertainty regarding the optimal dosing regimen for co-trimoxazole, especially given inter-regional variations in the reported susceptibility of local strains and the pharmacokinetics and pharmacodynamics within different populations. A recent pharmacokinetic/pharmacodynamic modelling study has suggested that previous recommendations may have resulted in underdosing of melioidosis patients in northeast Thailand and supported the use of a weight-based regimen [44].

Co-trimoxazole alone has been used for several years for the eradication phase of treatment in northern Australia with apparently excellent results [45]. In 2013, a group from southern Thailand published their experience of using co-trimoxazole alone, with similarly good results [46]. In a retrospective review over a 10-year period, only 1 (3.2%) of 31 patients who received co-trimoxazole alone relapsed compared with 5 (4.6%) of 109 treated with co-trimoxazole plus doxycycline. There was a considerably higher rate (25.7%) of gastrointestinal side effects in the latter group compared with the group receiving co-trimoxazole alone (6.5%), and only 83.5% of the patients receiving the combination regimen were able to complete ≥20 weeks of treatment as opposed to all those treated with co-trimoxazole alone.

More recently still, the results of a large, multicentre study (the MERTH study) conducted in north-east Thailand have been published [47]. This study enrolled 626 patients of whom 315 were assigned to receive co-trimoxazole plus doxycycline and 311 to receive co-trimoxazole plus placebo for a minimum of 20 weeks (extended beyond this on clinical grounds in 6% and 4% of cases, respectively). Forty patients (6%) with mild and localised melioidosis were treated with oral antibiotics only, whilst the others had received prior parenteral treatment, which in 357 cases (57%) had been given for >2 weeks. During more than 1100 person-years of follow-up, no differences were found between the groups in the rates of culture-confirmed (7% vs. 5%) or clinically suspected (3% in both groups) recurrences, or mortality, whether related to melioidosis (1% vs. 3%) or overall (8% vs. 6%). On the other hand, the risk of adverse events that required a switch of treatment (to co-amoxiclav) was significantly higher in the doxycycline group (59 patients; 19%) than in the placebo group (37 patients; 12%). Adverse drug reactions (mainly skin rashes, allergic reactions and gastrointestinal disorders) were reported in 39% of the placebo group and 53% of the doxycycline group overall and were classified as serious adverse events (e.g. Stevens–Johnson syndrome, severe hyponatraemia and severe hyperkalaemia) in 2% and 3%, respectively. Recurrent melioidosis did occur in as many as 10% of cases who were followed for 3 years after enrolment, however, although genotyping of paired primary and recurrent isolates in 29 cases suggested that 15 (52%) were actually due to re-infection, which tended to occur later [median 29 months; interquartile range (IQR) 13–37 months] than true relapse (median 7 months; IQR 6–13 months). The MERTH study at last confirms that the treatment of choice for the eradication phase of melioidosis treatment is co-trimoxazole monotherapy [48], except in the few patients in whom it is contraindicated or where the infecting isolate is co-trimoxazole-resistant. The optimum duration of the eradication phase remains to be determined, however, and further studies in Thailand are underway to address this question.

One outstanding issue relates to the true prevalence of co-trimoxazole resistance. Studies from Thailand have suggested that this may be as high as 18% of isolates [49] and yet the experience from other countries (Lao PDR, Australia, Cambodia) suggests that it may be considerably less common than this [50]. This may partly relate to the difficulty of measuring endpoints when testing co-trimoxazole against *B. pseudomallei* [51], [52]. It has been suggested that disk diffusion testing may be used as a screen to determine the susceptibility of *B. pseudomallei* for this combination and that MICs should be determined by Etest for any isolates that appear resistant or intermediate to co-trimoxazole by disk diffusion [49]. Unfortunately, no interpretive criteria for *B. pseudomallei* zone diameters have been published by the Clinical and Laboratory Standards Institute (CLSI), which recommends broth microdilution testing for *B. pseudomallei* against all antimicrobials [53]. Isolates with a MIC of >2 mg/L are classified as resistant, although no study has yet demonstrated a correlation between MICs and outcome in patients treated with co-trimoxazole alone.

### 2.3 Adjunctive treatment

Obviously patient management should always include the optimal supportive treatment for sepsis available, including maintenance of blood pressure, adequate glycaemic control, and management of respiratory and acute renal failure as well as drainage of abscesses where possible.

Since the overall mortality of patients with severe melioidosis remains high, and many of those who die do so in the first 48 h of treatment when antibiotics are unlikely to affect the outcome, various approaches have been tried to interrupt the inflammatory cascades and pathogenic processes that lead to death, or to augment host defences. The first such study involved the platelet-activating factor receptor antagonist lexipafant [54]. The study included 131 adult Thai patients with suspected sepsis who were randomised to receive lexipafant or placebo for up to 7 days, of whom 66 had positive blood cultures, 36 of which were *B. pseudomallei*. However, no differences were seen between the groups in either the mortality at 28 days or in any of the clinical or laboratory parameters measured. Steroids and activated protein C have also been used empirically in individual patients with sepsis caused by melioidosis but have never been evaluated prospectively and, as with sepsis in general, their role is unclear [55].

Neutrophils are thought to be important host effector cells in the control of *B. pseudomallei* infection. Considerable interest in the potential role of granulocyte colony-stimulating factor (G-CSF) in melioidosis followed the publication of a retrospective review of cases treated in northern Australia, which reported that the mortality rate of melioidosis patients with septic shock was reduced from 95% to 10% after the addition of G-CSF 300 μg/day for 10 days to standard treatment [56]. However, several other changes in patient management were made concurrently and so a prospective randomised controlled study was subsequently undertaken in Thailand [57]. In total 60 patients, 41 of whom had melioidosis confirmed by culture, were randomised to receive G-CSF 263 μg/day or placebo. Mortality rates were similar in both groups (G-CSF group 70% vs. placebo group 87%; risk ratio 0.81, 95% CI 0.61–1.06; *P* = 0.2), including patients with confirmed melioidosis (83% vs. 96%; *P* = 0.3). However, patients who received G-CSF survived longer than patients who received placebo (33 h vs. 18.6 h; hazard ratio = 0.56, 95% CI 0.31–1.00; *P* = 0.05). The authors concluded that although G-CSF itself had not been shown to reduce overall mortality in this relatively small study, it might ‘buy time’ for severely septic patients, allowing other measures such as correction of metabolic abnormalities and organ dysfunction that might ultimately improve survival. It is unlikely that definitive evidence to address this question will ever become available.

## 3 Prophylaxis (Table 3)

There are two main reasons for interest in prophylaxis against melioidosis: the possible exposure of laboratory workers to infection and the potential use of *B. pseudomallei* as a weapon by terrorists. Laboratory-acquired melioidosis has been reported, although there are only two well-described cases, both of which followed major lapses in laboratory technique [58], [59], and there is no evidence of infection occurring during simple manipulations such as identification and susceptibility testing. None the less, the organism is classified as a Hazard Group 3 pathogen and naturally anxiety occurs when laboratory staff are found to have inadvertently handled the organism on the open bench before the diagnosis is recognised.

As a result, international consensus recommendations were published in 2008 [60] and these were extended to the deliberate release situation in 2012 (Table 3) [16]. It should be made clear, however, that although there is some evidence of the partial effectiveness of post-exposure prophylaxis in animal models, particularly if given soon after exposure [61], the exposed animals frequently go on to develop symptomatic and progressive infection [62], and there is little reason to believe that antibiotic prophylaxis would prevent infection in humans.

## 4 Unanswered questions

### 4.1 Do all cases need parenteral treatment?

This is unclear. It is likely that mild, localised infection can be treated perfectly adequately with oral therapy alone, and indeed many individual cases have been managed in this way [47]. This specific question has, however, never been addressed in a clinical trial and may never be. In view of the potentially severe consequences of inadequate treatment, most experts recommend a precautionary approach including an initial parenteral phase for all except the very mildest superficial localised infections.

### 4.2 How long should each phase of treatment last in individual patients?

The evidence from clinical trials allows broad generalisations to be made, but because melioidosis is so variable in its manifestations, and patients with a diverse range of clinical forms of the disease are pooled together in the analysis of clinical trials, this does not allow specific recommendations to be made for individual patients. It is accepted that acute-phase treatment should be given for ≥10 days, as included in the study protocols of all clinical trials, but some patients who fail to defervesce or who have undrainable deep-seated abscesses may require longer periods of treatment. Recent analysis of data from melioidosis patients in Ubon Ratchathani has shown that having blood cultures taken at the end of the first and/or second week after hospitalisation that are positive for *B. pseudomallei* is strongly associated with death (adjusted odds ratio = 4.2, 95% CI 2.1–8.7, *P* < 0.001; and adjusted odds ratio = 2.6, 95% CI 1.1–6.0, *P* = 0.03, respectively), suggesting that blood cultures should be repeated weekly until negative in patients with bacteraemic melioidosis, and treatment extended if these are positive [63]. Even in the relatively controlled circumstances of a clinical trial such as the MERTH study, the duration of treatment given to individual patients varied considerably.

Similar uncertainties surround the precise duration of oral eradication treatment, although it is clear that this should usually be given for ≥12 weeks and potentially up to 20 weeks dependent on clinical progression [36]. In this latter study, the hazard ratio for relapse decreased by 29% for every 4-week increase in the duration of oral treatment. A study to compare 12 weeks versus 20 weeks of treatment with co-trimoxazole is underway in Thailand.

### 4.3 Are carbapenems better than ceftazidime?

There is as yet no hard evidence to support this, although several clinicians with experience of treating large numbers of cases of melioidosis believe that they may be. Workers in northern Australia have, since 1997, adopted meropenem 25 mg/kg every 8 h as their preferred treatment for selected patients with melioidosis, particularly those with severe sepsis, central nervous system infection, or relapse following ceftazidime treatment, with very favourable results (overall mortality 19% of 63 patients) [34]. There is a trial currently underway in Thailand comparing meropenem and ceftazidime, the results of which are eagerly awaited but which is currently on hold. For the time being, at least, there is no evidence that any agent is better than ceftazidime for reducing mortality during the acute phase of treatment. Unfortunately, the cost of ceftazidime remains difficult for many in endemic areas to afford: in Laos this is currently 29 000 Kip or US$3.62 per gram, so 10 days of treatment at full doses costs nearly US$220. Although this is now only ca. 17.5% of the 2012 Gross National Income per capita [64], as opposed to exceeding it when melioidosis in Laos was described as ‘Pandora's box’ a decade ago [65], this is still unaffordable for most people. Carbapenems are not currently available in Lao PDR and are even more expensive.

### 4.4 Should co-trimoxazole ever be added during the acute phase?

Despite the overall lack of clinical trial evidence of improved outcomes when co-trimoxazole is added to ceftazidime during acute-phase treatment, some experts still use the combination in specific circumstances, for example in the hope of achieving greater penetration of active agents into sites such as prostatic or other deep-seated abscesses or the central nervous system. It is unlikely that clinical trials of sufficient size will ever be conducted in these specific clinical settings and so this remains a matter for individual clinical judgement. It is certainly worth considering in patients who are deteriorating or failing to improve despite apparently adequate treatment.

### 4.5 What is the clinical significance of in vitro co-trimoxazole resistance?

The answer to this is that we really do not know. There are technical difficulties in assessing the susceptibility of *B. pseudomallei* to co-trimoxazole and this often has to be done by Etest or another method of measuring the MIC rather than by disk diffusion testing [52]. Isolated trimethoprim resistance related to increased expression of the BpeEF-OprC pump was found to be relatively common in clinical isolates of *B. pseudomallei* from Thailand and Australia, although these strains remained susceptible in vitro to co-trimoxazole [66]. Relatively high rates of co-trimoxazole resistance assessed by Etest (ca. 10–13%) have been reported in isolates of *B. pseudomallei* from Thailand [43], [49] and Malaysia [67], although the rate amongst isolates from the Lao PDR, Cambodia and Australia appears to be much lower than this [50]. In the comparative study of conventional eradication treatment with and without chloramphenicol [43], in vitro resistance to co-trimoxazole was not used as an exclusion criterion and there was no difference in relapse rate amongst those infected with co-trimoxazole-resistant strains, although the study was underpowered to detect the significance of this finding. Since it is not considered ethical to give co-trimoxazole monotherapy to patients with resistant isolates, however, alternative agents such as co-amoxiclav should be used for such patients.

### 4.6 Is acquired resistance a problem?

When the conventional regimen was being used extensively, acquired resistance to chloramphenicol, which was often accompanied by resistance to doxycycline and co-trimoxazole, was seen relatively frequently [5]. Resistance to the β-lactams now used for treatment has been seen to develop during treatment, usually related to mutations in the PenA β-lactamase gene that affects substrate specificity, but this is relatively uncommon, especially for carbapenems [68], [69], [70], [71], [72]. One unusual form of ceftazidime resistance recently reported in six patients involved the deletion of a gene encoding a penicillin binding protein 3 gene and was associated with growth defects that made the isolates difficult to culture in the laboratory [73]. Surprisingly high rates of resistance to ceftazidime (10%) and co-amoxiclav (30%) have recently been reported in isolates from Brazil [74]. Co-trimoxazole resistance may also emerge during treatment and so it is important to test isolates obtained during treatment for susceptibility, especially when this is associated with clinical deterioration. Further discussion of resistance mechanisms in *B. pseudomallei* can be found in a recent excellent review [75].

### 4.7 Are there any new treatments on the horizon?

Sadly the answer is ‘not really’. There are some drugs related to agents that are already used to treat melioidosis that show promising in vitro activity against *B. pseudomallei*, such as doripenem [76], [77], biapenem [78] and tebipenem, a carbapenem marketed in Japan of which an orally absorbed formulation is available [79]. Tigecycline is moderately active in vitro and has given some encouraging results in animal models but has yet to be evaluated in human melioidosis [77], [80], [81], [82]. The monosulfactam BAL30072 is some 2 log more active than ceftazidime and carbapenems against *B. pseudomallei* [83]. Various other classes of compounds, such as novel quinolones like sitafloxacin [84], COX-2 inhibitors [85], antimicrobial peptides [86], [87], [88], [89], [90], farnesol [91], CpG oligodeoxynucleotides [92], [93], glycogen synthase kinase inhibitors [94], sulfonylureas [95], cethromycin [96], plant extracts [97], [98], [99], snake venoms [100], methionine aminopeptidase inhibitors [101] and monoclonal antibody-based approaches [102], are under investigation for their in vitro activity against *B. pseudomallei* and therapeutic efficacy in animal models, but these are a long way from being used in human infections. These and other potential novel therapeutic approaches for melioidosis and glanders have recently been comprehensively reviewed [103], [104], [105].

## 5 Melioidosis treatment recommendations

International consensus recommendations for the treatment and prophylaxis of melioidosis and glanders were developed by an expert group that met in Australia in 2010 and have been published online [16]. These recommendations anticipated the results of the MERTH study and are reproduced with permission in Table 1, Table 2, Table 3. There has been no subsequent evidence published that would necessitate a change to these recommendations.

**Table 1:** Initial acute-phase therapy for melioidosis.^a^

Patient	Drug	Dosage/route	Frequency
With no complications	Ceftazidime	50 mg/kg (up to 2 g) intravenous	Every 8 h, or 6 g/day by continuous infusion after a 2 g bolus
With neuromelioidosis or persistent bacteraemia or in intensive care unit	Meropenem	25 mg/kg (up to 1 g) intravenous	Every 8 h

a Duration of acute-phase therapy is generally 10–14 days; however, >4 weeks of parenteral therapy may be necessary in cases of more severe disease, e.g. septic shock, deep-seated or organ abscesses, extensive lung disease, osteomyelitis, septic arthritis or neurological melioidosis. Consider adding trimethoprim/sulfamethoxazole (co-trimoxazole) for patients with severe infection involving the brain, prostate or other privileged site (same dosing as described for eradication therapy. Can be administered by intravenous infusion over 30–60 min every 12 h, or nasogastric, or oral, as appropriate). If co-trimoxazole is included, continue for the entire duration of the acute phase. Switching to meropenem is indicated if patient condition worsens while receiving ceftazidime, e.g. organ failure, development of a new focus of infection during treatment, or if repeat blood cultures remain positive. Depending on the severity of infection, the dose for patients >3 months can be <40 mg/kg (not to exceed 2 g/dose).

**Table 2:** Oral eradication-phase therapy for melioidosis.^a^

Drug	Patient characteristics	Recommended dosage/frequency
Trimethoprim/sulfamethoxazole (co-trimoxazole)^b^	Adult, >60 kg	160 mg/800 mg tablets; two tablets every 12 h
	Adult, 40–60 kg	80 mg/400 mg tablets; three tablets every 12 h
	Adult, <40 kg	160 mg/800 mg tablets; one tablet every 12 h OR
		80 mg/400 mg tablets; two tablets every 12 h
	Child	8 mg/40 mg per kg; maximum dose 320 mg/1600 mg every 12 h
OR		
Amoxicillin/clavulanic acid (co-amoxiclav)	Adult, >60 kg	500 mg/125 mg tablets; three tablets every 8 h c
	Adult, <60 kg	500 mg/125 mg tablets; two tablets every 8 h c
	Child	20 mg/5 mg per kg every 8 h; maximum dose 1000 mg/250 mg every 8 h

a Recommended duration of therapy is a minimum of 12 weeks.

b If the organism is susceptible and the patient does not have a documented allergy to it, oral co-trimoxazole is the agent of first choice. If the organism is resistant to co-trimoxazole or the patient is intolerant, the second-line choice is co-amoxiclav. Co-amoxiclav is available in different ratios and formulations depending on the source country. Co-amoxiclav at a ratio of 4:1 is preferred to ensure there is sufficient clavulanic acid [20].

c Weight-based dosage based on 20 mg/5 mg per kg per dose.

**Table 3:** Post-exposure prophylaxis for melioidosis.^a^

Drug	Patient characteristics	Recommended dosage/frequency
Trimethoprim/sulfamethoxazole (co-trimoxazole)	Adult, >60 kg	160 mg/800 mg tablets; two tablets every 12 h
	Adult, 40–60 kg	80 mg/400 mg tablets; three tablets every 12 h
	Adult, <40 kg	160 mg/800 mg tablets; one tablet every 12 h OR
		80 mg/400 mg tablets; two tablets every 12 h
	Child	8 mg/40 mg per kg; maximum dose 320 mg/1600 mg every 12 h
OR		
Amoxicillin/clavulanic acid (co-amoxiclav)	Adult, >60 kg	500 mg/125 mg tablets; three tablets every 8 h b
	Adult, <60 kg	500 mg/125 mg tablets; two tablets every 8 h b
	Child	20 mg/5 mg per kg every 8 h; maximum dose 1000 mg/250 mg every 8 h

a Duration of post-exposure prophylaxis is 21 days. If the organism is susceptible and the patient does not have a documented allergy to it, oral co-trimoxazole is the agent of first choice. If the organism is resistant to co-trimoxazole or the patient is intolerant, the second-line choice is co-amoxiclav.

b Weight-based dosage based on 20 mg/5 mg per kg per dose.
